# A positive association between the prevalence of circadian syndrome and overactive bladder in United States adults

**DOI:** 10.3389/fpubh.2023.1137191

**Published:** 2023-08-10

**Authors:** Yunfei Xiao, Shan Yin, Jiahao Wang, Jianwei Cui, Zhenzhen Yang, Jia Wang, Yunjin Bai

**Affiliations:** ^1^Department of Urology, Institute of Urology, West China Hospital, Sichuan University, Chengdu, China; ^2^Department of Urology, Affiliated Hospital of North Sichuan Medical College, Nanchong, China; ^3^Department of Clinical Laboratory, Nanchong Central Hospital, Nanchong, China

**Keywords:** overactive bladder, circadian syndrome, nocturia, urge urinary incontinence, NAHNES

## Abstract

**Objective:**

To explore the association between the prevalence of circadian syndrome (CircS) and overactive bladder (OAB).

**Materials and methods:**

Cross-section analysis was based on the National Health and Nutrition Examination Survey 2005–2018. Data regarding OAB was collected from questionnaires. The association between the prevalence of CircS and OAB was elucidated using three multivariable logistic regression models. Stratified and interaction analyses were performed to find whether some factors can modify the association.

**Results:**

Totally 8,033 males and 8,065 females were included. People with CircS had a significantly higher prevalence of OAB compared to the non-CircS group in the fully-adjusted model (OR = 1.238, 95%CI 1.080–1.419). A significant positive correlation between the number of CircS components and the prevalence of OAB was observed when the components were ≥ 6 (OR = 1.975, 95%CI 1.463–2.665). No significant interaction was seen in the three models.

**Conclusion:**

There is a positive association between the prevalence of CircS and OAB. When the number of components is ≥6, the prevalence of OAB shows a strongly positive correlation with the number of CircS components.

## 1. Introduction

Overactive bladder (OAB) is one of the common functional bladder disorders, and urinary urgency is defined as the core symptom. OAB is often accompanied by increased daytime frequency and/or nocturia, with urinary incontinence or without, in the absence of urinary tract infection or other detectable disease (1). With social stress increasing and lifestyle habits changing, the prevalence of OAB has risen in recent years (2). The five-country European Prospective Investigation into Cancer and Nutrition involving more than 19,000 adults demonstrates that the overall prevalence of OAB is 11.8% and is more common in women than in men (12.8 vs. 10.8%) (3). A study regarding OAB on physical occupational limitations shows the prevalence of OAB is around 30% in American women (4). In addition, the symptoms of OAB more easily occur, especially in a tense environment, which interferes with daily life and worsens the quality of life.

For OAB patients, the hallmark urodynamic feature is detrusor overactivity, and other forms of urethra–bladder dysfunction account for a small percentage. Although some theories are promoted to explain the pathophysiology of OAB, no identified doctrine has been established and universally accepted till now. Given the unclear etiology, long-term comprehensive treatment is proposed for most individuals, and life habit changes, regular local exercise, drugs (vaginal estrogen, anticholinergics, and β3 agonists), and invasive treatment (sacral neuromodulation, percutaneous tibial nerve stimulation, and surgery) are all considered (5). However, the treatments may only relieve the symptoms and are still substantially incapable of curing OAB.

With the awareness of internal biological rhythm raised, circadian syndrome (CircS) was proposed as a more reasonable cluster for metabolic syndrome (MetS) and its comorbidities (6). The CircS is present with any four of the following disorders, including dyslipidemia [triglycerides raising or high-density lipoprotein cholesterol decreased (HDL-C)], hypertension, central obesity, diabetes, depression, and short sleep. If a person is exposed to an unhealthy status in the long term, the circadian rhythm is vulnerable and more easily disturbed (7). Recent research has proposed that MetS and its comorbidities (sleep deprivation and depression) make a significant effect on the prevalence of OAB. In addition, mounting evidence shows that CircS is correlated with cardiovascular disease (CVD), lower urinary tract symptoms, and kidney stones (8–10). All of these factors mentioned above are not only closely related to internal biological clocks, but also are the risk factors for OAB (11, 12). Therefore, whether a relationship between the prevalence of CircS and OAB exists is worth exploring.

To the best of our knowledge, there is no study regarding the association between the prevalence of CircS and OAB. Thus, this cross-section study was established by analyzing the large population data from the National Health and Nutrition Examination Survey (NHANES). We hypothesize that CircS is positively associated with the prevalence of OAB.

## 2. Methods

### 2.1. Study design and population

**Figure 1:**
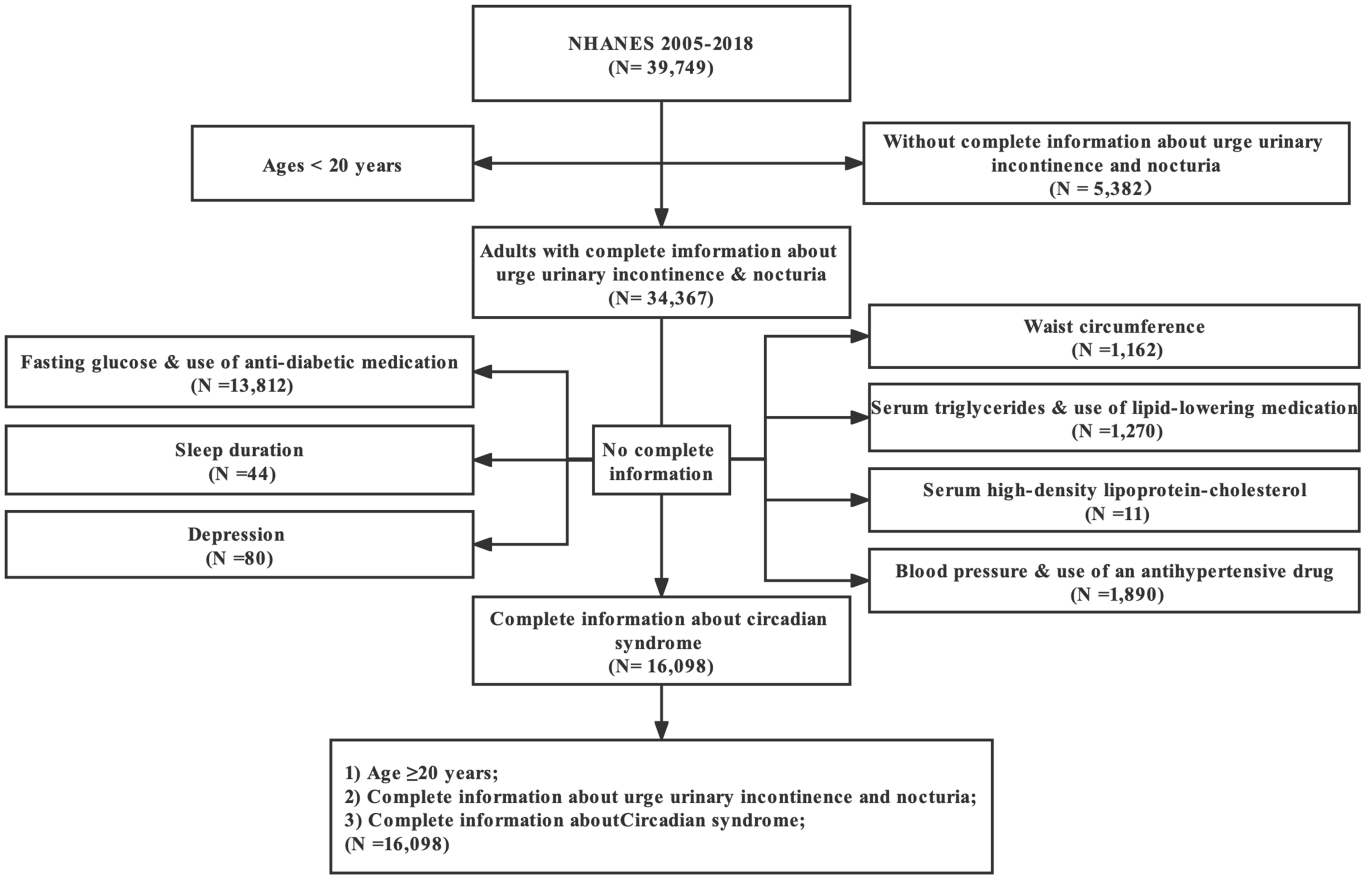
Flow diagram of obtaining the final inclusion in the population.

All of the data analyzed here were cited from the NHANES 2005–2018 (seven cycles: 2005–2006, 2007–2008, 2009–2010, 2011–2012, 2013–2014, 2015–2016, and 2017–2018), which were conducted by the Center for Disease Control and Prevention (CDC). This database mainly consisted of a series of cross-sectional, nationally representative surveys involving civilian, non-institutionalized American adults, and was proposed to measure the health and nutritional status of American adults and children. In addition, NHANES was updated every 2 years as planned and designed to be accessible to researchers for free. There were 39,749 participants enrolled initially. First, we excluded 5,331 people without complete information about UUI, and 51 participants with absent information of nocturia frequency. Moreover, 18,269 people with missing data for CircS diagnosis were excluded. Finally, 16,098 participants were admitted (details in Figure 1).

All NHANES study protocols were approved by the Ethics Review Committee of the NCHS, and consent was obtained from all participants.

### 2.2. Diagnosis of overactive bladder

**Figure 2:**
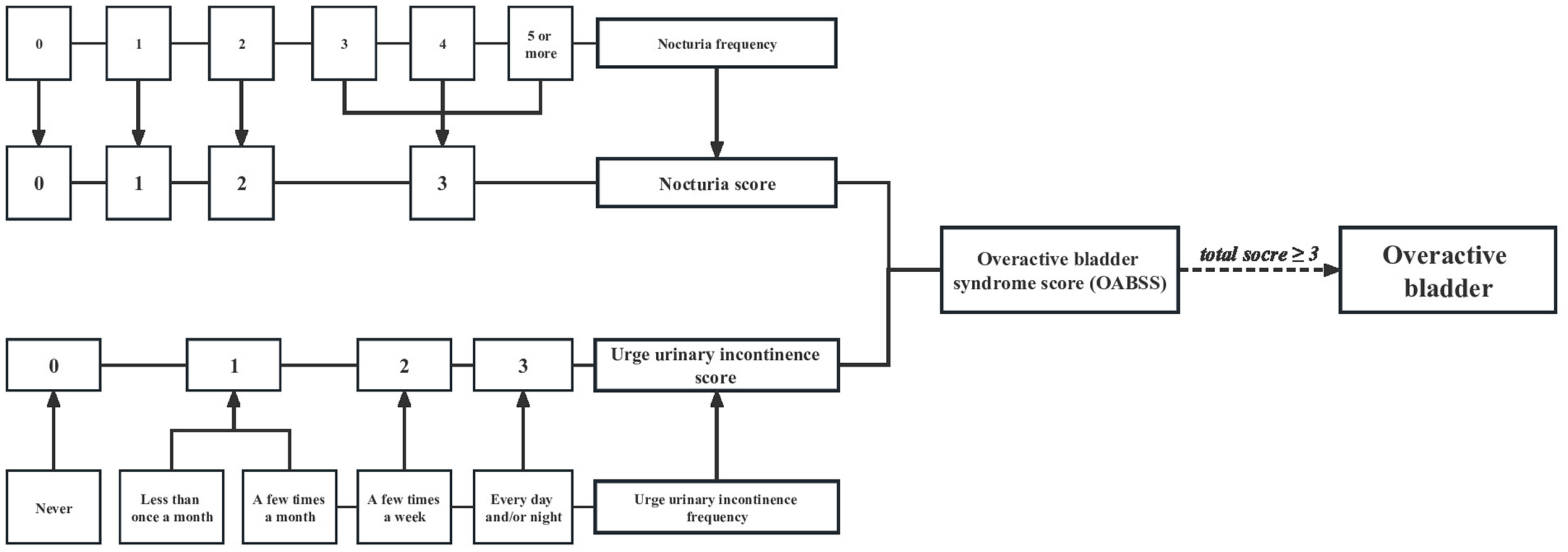
Flow diagram of the overactive bladder diagnosis based on overactive bladder syndrome score.

Overactive bladder was defined as an overactive micturition reflex, which was marked by urge urinary incontinence (UUI) and nocturia. All of the information acquired here was based on the questionnaires, which were completed by trained research staff through face-to-face interviews. UUI was obtained with the question “During the past 12 months, have you leaked or lost control of even a small amount of urine with an urge or pressure to urinate and you could not get to the toilet fast enough?,” and the severity was evaluated by the question “How frequently does this occur?.” Moreover, the gravity of nocturia was assessed with the question “During the past 30 days, how many times per night did you most typically get up to urinate, from the time you went to bed at night until the time you got up in the morning.” In addition, OAB was qualified with the aid of the overactive bladder symptom score (OABSS). The details were described in Figure 2. A participant with an overall OABSS ≥3 was considered to suffer from OAB.

### 2.3. Diagnosis of circadian syndrome

As the main exposure variable, CircS was diagnosed when a person had ≥4 of these components: (1) central obesity: women (men) with a waist circumference ≥ 88 cm (≥ 102 cm); (2) elevated triglycerides (≥150 mg/dL) or use of lipid-lowering medication; (3) decreased HDL-C (<40 mg/dL in men and < 50 mg/dL in women) or use of lipid-lowering medication; (4) elevated blood pressure (systolic ≥130 or diastolic ≥85 mmHg) or use of an antihypertensive drug; (5) elevated fasting glucose (≥100 mg/dL) or use of anti-diabetic medication; (6) reduced sleep duration (≤6 h/day); and (7) depression symptoms defined by patient health status questionnaire-9 (8).

### 2.4. Covariates

The information on covariates was collected through questionnaires, examinations, and laboratory tests. The continuous variables included age (≥20 years), poverty income ratio (PIR), body mass index (BMI), estimated glomerular filtration rate (eGFR), and healthy eating index-2015 (HEI-2015). The categorical variables were listed as gender, ethnicity (Mexican American, Non-Hispanic white, Non-Hispanic black, and Other ethnicity), education level (less than 9th grade, 9–11th grade, high school graduate, some college, college graduate, or above), marital status (married, widowed, divorced, separated, never married, and living with a partner), smoking (never, former, and now), and alcohol, cancer, stroke, CVD, moderate activity, and vigorous activity (all no/yes). Missing data were present in alcohol, moderate activity, and vigorous activity.

### 2.5. Statistical analysis

Proportions and mean ± standard errors (SE) were used to express the baseline characteristics. Chi-square analysis was used to adjust comparisons of categorical variables, while linear regression was used to correlate continuous variables. To avoid potential bias due to missing data in the covariates of alcohol, moderate and vigorous activity, the absent parts were established as dummy variables.

The participants were categorized into two groups by CircS diagnosis, and three multivariable logistic models were built to analyze the association between CircS and OAB. Additionally, the correlation between the number of CircS components and OAB was also analyzed. In the non-adjusted model, no factor was adjusted. In the minimally-adjusted model, age and ethnicity were adjusted. For the fully-adjusted model, age, ethnicity, gender, education, BMI, marital status, PIR, HEI-2015, smoking, vigorous activity, moderate activity, alcohol, stroke, CVD, eGFR, and cancer were all adjusted. To make a deep analysis of the association, stratified and interaction analyses were also performed regarding all potential confounding factors above.

For all analyses, the sample weights of the seven continuous cycles were combined according to the method recommended by the CDC.^1^ The essential work made the sample representative of the civilian population of the United States and produced an unbiased national estimate. Statistical analyses were all performed on R 4.0 (http://www.R-project.org; the R Foundation) and EmpowerStats (http://www.empowerstats.com; X&Y Solutions, Inc.). Differences were considered statistically significant when *p* was less than 0.05.

## 3. Results

### 3.1. Baseline characteristics of the population

From the seven cycles of NHANES (2005–2018), 16,098 participants were involved eventually. Then the participants were divided into two groups by CircS, including 5,943 people with CircS and 10,155 people without it. Significant differences were present in a majority of the baseline characteristics (Table 1). Females constituted most of the participants with OAB (61.46%, Supplementary Table S1). Compared to the non-CircS group, people with CircS were older (mean ± SE, 58.11 ± 0.31 years) and tended to have a higher BMI (33.07 ± 0.14 kg/m^2^). As expected, a larger proportion of the people with CircS had UUI (31.72%), nocturia (77.46%), and OAB (28.47%). Additionally, these people were more likely to have general education (less than college, 47.07%), solitary status (widowed, divorced, separated, and never married, 35.14%), cancer (16.42%), CVD (21.96%), stroke (6.67%), and smoking (52.86%). However, an opposite trend was seen in the lower PIR (2.86 ± 0.04), eGFR (84.51 ± 0.45 mL/min/1.73m^2^), proportions of moderate activity (34.99%), HEI-2015 (50.51 ± 0.27), vigorous activity (17.04%), and alcohol (59.42%). Moreover, each component of CircS appeared more frequently in the OAB group, such as central obesity (76.55%), elevated serum triglycerides (58.97%), reduced serum HDL-C (59.11%), hypertension (47.73%), elevated plasma glucose (69.37%), short sleep (16.18%), and depression (16.69%). Additionally, Table 2 presented the details of OABSS. Notably, people with CircS were linked with higher UUI scores (0.43 ± 0.01), nocturia scores (1.35 ± 0.02), and OABSS (1.78 ± 0.03).

**Table 1:** Characteristics of participants by categories of circadian syndrome: NHANES 2005–2018^*^.

Variables	*N*	All (*n* = 16,098)	Groups		*p* value
			Non-circadian syndrome (*n* = 10,155)	Circadian syndrome (*n* = 5,943)	
Age (years, mean ± SE)		48.76 ± 0.26	44.17 ± 0.30	58.11 ± 0.31	<0.001
20–34 (%)	3,664	24.81	33.67	6.75	
35–49 (%)	3,735	26.56	29.61	20.35	
50–64 (%)	4,477	28.10	23.79	36.87	
≥65 (%)	4,222	20.53	12.93	36.03	
PIR (mean ± SE)		3.01 ± 0.04	3.09 ± 0.04	2.86 ± 0.04	<0.001
≤1.3 (%)	4,602	20.72	19.65	22.90	
>1.3 and ≤ 3.5 (%)	5,697	36.73	35.51	39.21	
>3.5 (%)	4,473	42.56	44.83	64.65	
BMI (kg/m^2^, mean ± SE)		29.32 ± 0.10	27.49 ± 0.11	33.07 ± 0.14	<0.001
<25 (%)	4,346	28.32	38.26	7.99	
25–30 (%)	5,307	32.71	35.33	27.36	
≥30 (%)	6,398	38.96	26.41	64.65	
eGFR (mL/min/1.73m^2^, mean ± SE)		94.00 ± 0.33	98.58 ± 0.39	84.51 ± 0.45	<0.001
HEI-2015 (mean ± SE)		50.51 ± 0.23	50.75 ± 0.27	50.51 ± 0.27	0.022
Gender (%)					0.301
Female	8,033	49.80	50.18	49.04	
Male	8,065	50.20	49.82	50.96	
Education (%)					<0.001
Less than 9th grade	1,754	5.56	4.43	7.85	
9–11th grade	2,324	10.71	9.85	12.47	
High school graduate	3,697	23.27	21.56	26.75	
Some college	4,696	31.33	31.04	31.93	
College graduate or above	3,616	29.13	33.12	20.99	
Ethnicity (%)					<0.001
Mexican American	2,588	8.51	8.91	7.69	
Other Hispanic	1,609	5.46	5.70	4.97	
Non-Hispanic white	6,927	68.52	67.43	70.74	
Non-Hispanic black	3,324	10.41	10.43	10.37	
Other ethnicity	1,650	7.10	7.52	6.23	
Marital (%)					<0.001
Married	8,492	56.61	55.30	59.28	
Widowed	1,337	5.95	3.76	10.42	
Divorced	1,782	10.48	9.18	13.14	
Separated	549	2.28	2.34	2.16	
Never married	2,665	16.62	20.15	9.42	
Living with a partner	1,268	8.06	9.27	5.58	
Cancer (%)					<0.001
No	14,502	89.55	92.48	83.58	
Yes	1,586	10.45	7.52	16.42	
Smoking (%)					<0.001
Never	8,652	53.47	56.57	47.14	
Former	4,228	26.89	23.16	34.49	
Now	3,208	19.65	20.28	18.37	
Alcohol (%)					<0.001
No	3,992	19.77	17.68	24.03	
Yes	9,892	65.04	67.80	59.42	
Missing	2,214	15.19	14.53	16.56	
Stroke (%)					<0.001
No	15,383	96.73	98.39	93.33	
Yes	697	3.27	1.61	6.67	
Vigorous activity (%)					<0.001
No	11,412	68.39	66.35	72.55	
Yes	2,766	18.75	19.59	17.04	
Missing	1,920	12.85	14.05	10.41	
Moderate activity (%)					<0.001
No	8,909	50.55	48.57	54.60	
Yes	5,267	36.85	37.37	34.99	
Missing	1,922	12.86	14.06	10.41	
CVD (%)					<0.001
No	14,056	89.75	95.49	78.04	
Yes	2,041	10.25	4.51	21.96	
Central obesity (%)					<0.001
No	6,479	41.02	56.18	10.11	
Yes	9,619	58.98	43.82	89.89	
Elevated serum triglycerides (%)					<0.001
No	8,652	56.46	79.13	10.22	
Yes	7,446	43.54	20.87	89.78	
Reduced serum HDL-C (%)					<0.001
No	8,613	56.25	77.86	12.21	
Yes	7,485	43.75	22.14	87.79	
Hypertension (%)					<0.001
No	9,514	64.48	78.44	36.01	
Yes	6,584	35.52	21.56	63.99	
Elevated plasma glucose (%)					<0.001
No	6,445	43.90	60.88	9.28	
Yes	9,653	56.10	39.12	90.72	
Short sleep (%)					<0.001
No	13,805	88.05	92.07	79.86	
Yes	2,293	11.95	7.93	20.14	
Depression (%)					<0.001
No	14,669	92.37	95.71	85.57	
Yes	1,429	7.63	4.29	14.43	
UUI frequency (%)					<0.001
Never	12,195	78.82	83.98	68.28	
Less than once a month	1,710	10.04	8.51	13.18	
A few times a month	1,258	6.59	4.74	10.38	
A few times a week	563	2.79	1.89	4.62	
Every day and/or night	363	1.76	0.88	3.54	
Nocturia frequency (%)					<0.001
0	4,673	33.24	38.46	22.54	
1	5,925	39.65	40.25	38.43	
2	3,173	17.42	14.52	23.38	
3	1,490	7.11	5.12	11.21	
4	490	2.04	1.37	3.42	
5 or more	109	0.54	0.29	1.03	
Overactive bladder (%)					<0.001
No	12,460	82.45	87.81	71.53	
Yes	3,638	18.55	12.19	28.47	

* For continuous variables, the *t*-test for slope was used in generalized linear models.

Values are expressed as mean + SE or %. SE, standard error, BMI, body mass index; PIR, poverty income ratio; HEI-2015, healthy eating index-2015; CVD, cardiovascular disease; UUI, urge urinary incontinence; OABSS, overactive bladder symptom score; eGFR, estimated glomerular filtration rate.

**Table 2:** Overactive bladder syndrome score categorized by circadian syndrome.

Variables	*N*	All (*n* = 16,098)	Groups		*p* value
			Non-circadian syndrome (*n* = 10,155)	Circadian syndrome (*n* = 5,943)	
Urge urinary incontinence score (mean ± SE)		0.27 ± 0.01	0.20 ± 0.01	0.43 ± 0.01	<0.001
0 (%)	12,204	78.83	83.98	68.31	
1 (%)	2,968	16.63	13.24	23.53	
2 (%)	563	2.79	1.89	4.63	
3 (%)	363	1.75	0.88	3.53	
Nocturia score (mean ± SE)		1.05 ± 0.01	0.91 ± 0.01	1.35 ± 0.02	<0.001
0 (%)	4,673	32.94	38.21	22.21	
1 (%)	5,925	39.29	39.99	37.87	
2 (%)	3,173	17.26	14.42	23.04	
3 (%)	2,327	10.51	7.38	16.88	
Overactive bladder symptom score (mean ± SE)		1.33 ± 0.02	1.11 ± 0.02	1.78 ± 0.03	<0.001
0 (%)	4,069	29.14	34.60	17.99	
1 (%)	5,183	34.85	36.64	31.22	
2 (%)	3,208	18.46	16.57	22.32	
3 (%)	2,338	11.75	9.00	17.37	
4 (%)	856	3.90	2.32	7.11	
5 (%)	266	1.20	0.62	2.41	
6 (%)	178	0.69	0.25	1.59	
Overactive bladder (%)					<0.001
No	12,460	82.45	87.81	71.53	
Yes	3,638	17.55	12.19	28.47	

^*^For continuous variables, the *t*-test for slope was used in generalized linear models. Values are expressed as mean + SE or %. SE, standard error.

### 3.2. Multivariate regression analysis

We made a multivariate regression analysis and listed the effect sizes of association between CircS and OAB in Table 3. Results showed that the CircS group was associated with a higher prevalence of OAB in the non-adjusted model (OR = 2.868, 95%CI 2.582–3.185, *p* < 0.001). The same trend was seen both in the minimally-adjusted model (OR = 1.810, 95%CI 1.607–2.039, <0.001) and the fully-adjusted model (OR = 1.238, 95%CI 1.080–1.419, *p* = 0.003). Moreover, multivariate logistic regression analysis was conducted under stratified conditions, and a significant positive correlation between the number of CircS components and the prevalence of OAB was observed when the number of components was ≥6 (OR = 1.975, 95%CI 1.463–2.665, *p* ≤ 0.001).

**Table 3:** Association of circadian syndrome with the prevalence of overactive bladder.

Variables	Non-adjusted model^*^		Minimally-adjusted model^**^		Fully-adjusted model^***^	
	OR (95%CI)	*p*	OR (95%CI)	*p*	OR (95%CI)	*p*
Circadian syndrome						
No	Ref		Ref		Ref	
Yes	2.868 (2.582, 3.185)	<0.001	1.810 (1.607, 2.039)	<0.001	1.238 (1.080, 1.419)	0.003
Components of circadian syndrome						
4	Ref		Ref		Ref	
5	1.218 (1.033, 1.437)	0.021	1.077 (0.907, 1.280)	0.399	0.972 (0.809 1.167)	0.756
≥6	2.907 (2.288, 3.693)	<0.001	2.672 (2.068, 3.452)	<0.001	1.975 (1.463, 2.665)	<0.001

CI, confidence interval; OR, odds ratio. ^*^Non-adjusted model adjusts for none. ^**^Minimally-adjusted model adjusts for age, ethnicity. ^***^Fully-adjusted model adjusts for age, gender, ethnicity, education, BMI, marital, PIR, HEI-2015, smoking, alcohol, vigorous activity, moderate activity, stroke, CVD, eGFR, and cancer.

### 3.3. Stratified and interaction analyses

**Figure 3:**
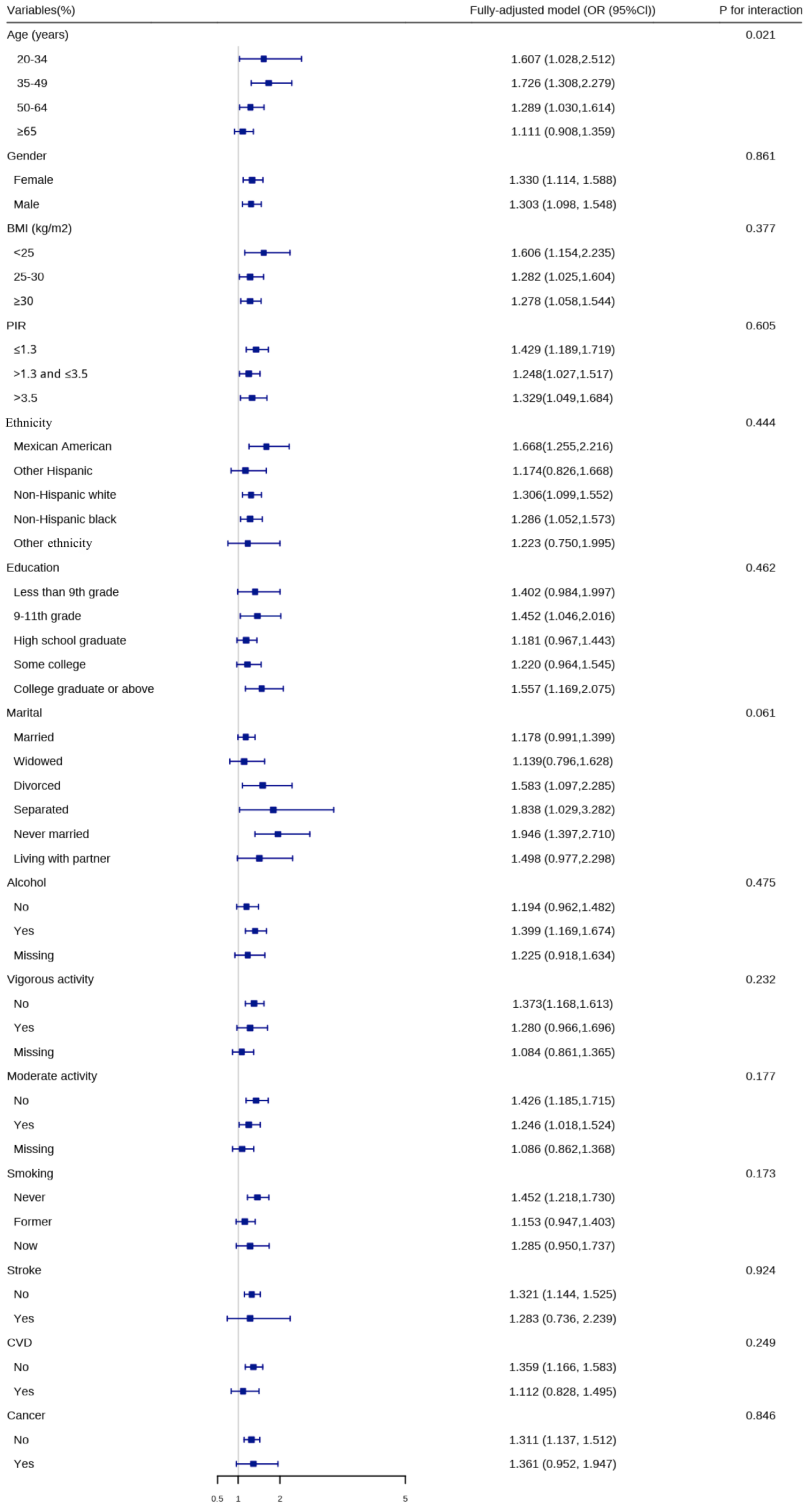
Logistic regression analyses to identify variables that modify the correlation between circadian syndrome and overactive bladder.

To further elucidate the association between the prevalence of CircS and OAB, we performed interaction analysis in the fully-adjusted model (Figure 3). We found that the association between the two was modified by age (*p* = 0.021) for interaction. In other words, when people were in the aged phase of 20–34, 35–49, 50–64, or ≥ 65 (OR: 1.607, 1.726, 1.289, 1.111), the positive association was more obvious. Nevertheless, the direction of effect values was consistent across subgroups. No more interaction was indicated among other factors.

## 4. Discussion

This study demonstrates a positive association between the prevalence of CircS and OAB, and the number of CircS components is also significantly related to a higher prevalence of OAB when the number is ≥6. Additionally, the positive association is modified by age, but the direction of effect values is consistent across subgroups.

Although numerous works have been done around the associations of OAB with MetS, depression, and sleep deprivation, these factors are always analyzed alone and never considered at the same time. Therefore, the first work to show the association between CircS and OAB was conducted here. With changes in life pace and lifestyle, the prevalence of CircS has increased annually (7). CircS depends primarily on the dysfunction of circadian rhythm, which is a set of biological variables (with about a 24-h period) produced by circadian clocks (13). Circadian rhythm can affect not only macroscopic organisms but also various organs and cells, making it one of the most important and fundamental features of biological activities (14). When the circadian rhythm in the body is abnormally altered, many problems are supposed to occur in the organism, including inflammatory, immune, and metabolic disorders. All of these changes contribute to OAB development. Moreover, recent evidence demonstrates that circadian clocks are governed by the suprachiasmatic nucleus in the hypothalamus regardless of peripheral clocks (15). The master body clock is accompanied by light intensity and duration, and the clock becomes unstable with the lifestyle change (16). Unfortunately, no prior research has yet focused on the association between light or shift work and the prevalence of OAB. Thus, this hypothesis still shall be elucidated with further evidence, which is what we plan to do in future studies to deeply explore the mechanism to support our results directly.

Furthermore, there is also some evidence to support our results indirectly. CircS is a cluster of diverse psychological and physiological impairments, including MetS (central obesity, elevated serum triglycerides, reduced serum HDL-C, elevated plasma glucose, and HBP), depression, and short sleep. Previous studies reveal that OAB is more prevalent in people with MetS than those without MetS (17). Moreover, higher risk and intensity of OAB were both observed in the population with psychological diseases, such as depression and anxiety (18). On the flip side, incontinence in public makes people fear, so they always avoid social interaction, which further exacerbates the severity of depression. Thus, a vicious circle is formed. Additionally, insomnia increases the odds of nocturia in some ways. First, most of these patients develop bad habits, such as drinking a large quantity of water before bedtime or drinking stimulating drinks at night including tea, coffee, and alcohol (19, 20). Second, people with insomnia are often accompanied by anxiety, which is worsened upon waking at night and causes reflex hyperactivity of the detrusor, leading to the appearance of OAB (21). For those patients with severe insomnia, who often feel difficultly falling asleep within a short time after waking up at night, they make a habit of urination, whether or not they have symptoms of urinary urgency. Jooyeon et al. revealed that urinary symptoms were improved significantly with great sleep efficiency via a biofeedback-based sleep improvement program (22).

In recent studies, several potential mechanisms were also proposed to explain the association between CircS and the prevalence of OAB. Autonomic hyperactivity plays an essential role. Compared to people without it, overactivated sympathetic activity and decreased vagal activity are more likely to be seen in people with MetS and depression (18, 23). In a long-term fructose-induced metabolic syndrome model, the expressions of acetylcholine and muscarinic cholinergic receptors were upregulated (24, 25). Additionally, the risk of atherosclerosis and CVD was raised when people suffered from CircS (8). The vascular alternation led to less blood supply to the bladder and bladder ischemia, which contributed to the occurrence of OAB. Apart from that, endoplasmic reticulum stress, autophagy, and apoptosis of the bladder were enhanced in the rat model of pelvis ischemia (26). For men with benign prostate enlargement, intravesical pressure increased due to enhanced contraction of the detrusor muscle as a result of bladder outlet obstruction, making bladder ischemia more pronounced (27). In addition to the gut microbiota, evidence supported that urine microbiota also affected urinary diseases, including OAB, kidney stones, and bladder cancer (28, 29). When circadian rhythms were disturbed, the microbiota in the urine was altered and its decreased abundance resulted in dysbiosis of the urinary flora. This impaired the beneficial effects of urinary microbiota on the bladder regulation and protection (30, 31). Moreover, the bladder-gut-brain axis (BGBA) might play an indispensable role in the pathophysiology of OAB (32). In addition to frequent co-occurrence and the symptom overlap with intestinal disorders, the functional urological disorder was also related to neuroticism (33). Central sensitization was induced by some negative affectivity, such as depression, and anxiety. The exact mechanism of BGBA is complex and remains elusive, but the following factor may be involved. An increase in membrane excitability and synaptic efficacy resulted in central sensitization, leading to automatic nervous system imbalance (34). Ovarian hormone deficiency was more likely to attack people with CircS, which contributed to the development of OAB (35). In a rat animal experiment, the ovariectomized rats mimicking the physiological condition of menopause were subjected to OAB, and low-intensity extracorporeal shock wave therapy attenuated inflammatory response and inhibited interstitial fibrosis (36). Therefore, although many efforts have been made, more trials and experiments are still needed to draw some convening pathological and psychological explanations.

This study also reveals that age modifies the positive association between the prevalence of CircS and OAB, but the direction of effect values is consistent across subgroups. Previous studies reported that aging people were more prone to a higher prevalence of OAB, which disagrees with our results. The disrupted circadian rhythm made aging accelerate, and the aging process was also associated with the frailty of the circadian rhythm (37, 38). Moreover, previous studies certificated that the incidence of OAB increased with age. A clinical trial showed that the nocturnal polyuria index was upregulated significantly with age, but no difference existed in genders (39). Based on a rat study, it was reported that aging bladders exhibited an elevated release of intrinsic ATP, resulting in heightened stimulation of downstream targets (40). Despite the complexity of the mechanisms behind the phenomenon, the spontaneous rise of intracellular Ca^2+^ may serve as an underlying mechanism (41). However, contrary to expectations, no significant interaction was found in gender in the study. Therefore, prospective and comprehensive studies with larger populations are required to elaborate on the conflicting phenomenon, and this is our further work.

This study has both pros and cons. First, the national multi-ethnic survey based on NHANES in a large number of populations makes the study representative and applicable. Second, CircS is a new concept combined with biological rhythms that are more consistent with the majority of physiological processes. No study has focused on the association between the prevalence of CircS and OAB. However, some limitations still need to be mentioned. Owing to the cross-section analyses, the causal relationship between the prevalence of CircS and OAB cannot be elucidated. In addition, although the diagnosis of OAB was governed by the synthesized OABSS in our study, laboratory diagnostic tests were absent in NHANES. Third, the symptoms of nocturia and urinary incontinence were collected via questionnaires, recall bias was probable.

## 5. Conclusion

There is a positive association between the prevalence of CircS and OAB. Notably, CircS is positively correlated with a higher prevalence of OAB. Furthermore, individuals with six or more CircS components have a strong correlation with an increased likelihood of experiencing OAB. However, to gain a deeper understanding, it is necessary to conduct more comprehensive and prospective cohort studies due to the cross-sectional design.

## Data availability statement

The datasets presented in this study can be found in online repositories. The names of the repository/repositories and accession number(s) can be found at: https://www.cdc.gov/nchs/nhanes/index.htm, National Center for Health Statistics.

## Ethics statement

This study was performed using public data from the National Center for Health Statistics (NCHS) program and the National Health and Nutrition Examination Survey (NHANES). The data have been de-identified and not merged or augmented in a way that has compromised the privacy of the participants. Therefore, the study requires no further approval and follows ethical guidelines. Participant data were obtained from the publicly available NHANES, so no additional consent was obtained.

## Author contributions

YFX and SY: conception and design. JW and YJB: administrative support and supervision. YFX, JHW, JWC, and ZZY: collection and assembly of data. SY and JHW: data analysis and interpretation. YFX: manuscript writing. YFX, SY, JHW, JWC, ZZY, JW, and YJB: final approval of manuscript. All authors contributed to the article and approved the submitted version.

## Funding

This work was supported by the Key Research and Development Projects of Sichuan Science and Technology Department (grant number: 2020YFS0189 and 2022YFS0306), and the Project of Sichuan Provincial Health and Health Commission (grant number: 20PJ064).

## Conflict of interest

The authors declare that the research was conducted in the absence of any commercial or financial relationships that could be construed as a potential conflict of interest.

## Publisher’s note

All claims expressed in this article are solely those of the authors and do not necessarily represent those of their affiliated organizations, or those of the publisher, the editors and the reviewers. Any product that may be evaluated in this article, or claim that may be made by its manufacturer, is not guaranteed or endorsed by the publisher.

## Footnotes

^1^ https://www.cdc.gov/nchs/nhanes/index.htm
