# Supplementary material for: A positive association between the prevalence of circadian syndrome and overactive bladder in United States adults
Source: Front Public Health. 2023 Aug 10;11:1137191. doi: 10.3389/fpubh.2023.1137191 (PMC10449362; doi:10.3389/fpubh.2023.1137191)
Supplement: Supplementary file 1 [file Table_1.pdf]

Table S1 Characteristics of participants by categories of overactive bladder: NHANES 2005-2018*.

Variables	N	All (n=16,098)	Groups: Non-overactive bladder (n=12,460)	Groups: Overactive bladder (n=3,638)	P-value
Age (years, mean ± SE)		48.76 ± 0.26	46.71 ± 0.24	58.37 ± 0.37	<0.001
20-34 (%)	3,664	24.81	28.06	9.55	
35-49 (%)	3,735	26.56	28.60	16.96	
50-64 (%)	4,477	28.10	26.91	33.68	
≥65 (%)	4,222	20.53	16.43	39.81	
PIR (mean ± SE)		3.01 ± 0.04	3.11 ± 0.03	2.55 ± 0.06	<0.001
≤1.3 (%)	4,602	20.72	19.00	28.95	
>1.3 and ≤3.5 (%)	5,697	36.73	35.88	40.75	
>3.5 (%)	4,473	42.56	45.12	30.30	
BMI (kg/m^2, mean ± SE)		29.32 ± 0.10	28.83 ± 0.10	31.66 ± 0.19	<0.001
<25 (%)	4,346	28.32	30.48	18.18	
25-30 (%)	5,307	32.71	33.36	29.66	
≥30 (%)	6,398	38.96	36.16	52.16	
eGFR (mL/min/1.73m^2, mean ± SE)		94.00 ± 0.33	95.75 ± 0.33	85.72 ± 0.59	<0.001
HEI-2015 (mean ± SE)		50.51 ± 0.23	50.50 ± 0.24	50.54 ± 0.37	0.924
Gender (%)					<0.001
Female	8,033	49.80	47.32	61.46	

Male	8,065	50.20	52.68	38.54	
Education (%)					<0.001
Less than 9th grade	1,754	5.56	4.66	9.76	
9-11th grade	2,324	10.71	9.72	15.36	
High school graduate	3,697	23.27	22.83	25.35	
Some college	4,696	31.33	31.58	30.18	
College graduate or above	3,616	29.13	31.32	19.36	
Ethnicity (%)					<0.001
Mexican American	2,588	8.51	8.15	8.48	
Other Hispanic	1,609	5.46	5.47	5.41	
Non-Hispanic white	6,927	68.52	69.19	65.38	
Non-Hispanic black	3,324	10.41	9.29	15.67	
Other ethnicity	1,650	7.10	7.53	5.06	
Marital (%)					<0.001
Married	8,492	56.61	57.18	53.93	
Widowed	1,337	5.95	4.52	12.66	
Divorced	1,782	10.48	9.87	13.36	
Separated	549	2.28	2.08	3.19	
Never married	2,665	16.62	17.90	10.62	
Living with a partner	1,268	8.06	8.44	6.24	
Cancer (%)					<0.001
No	14,502	89.55	91.21	81.74	
Yes	1,586	10.45	8.79	18.26	
Smoking (%)					<0.001

Never	8,652	53.47	54.54	48.80	
Former	4,228	26.89	25.96	31.23	
Now	3,208	19.65	19.49	20.38	
Alcohol (%)					<0.001
No	3,992	19.77	18.49	25.78	
Yes	9,892	65.04	66.49	58.21	
Missing	2,214	15.19	15.02	16.01	
Stroke (%)					<0.001
No	15,383	96.73	97.64	92.40	
Yes	697	3.27	2.36	7.60	
Vigorous activity (%)					0.009
No	11,412	68.39	67.54	57.01	
Yes	2,766	18.75	19.34	15.97	
Missing	1,920	12.85	13.12	11.61	
Moderate activity (%)					<0.001
No	8,909	50.55	49.18	57.01	
Yes	5,267	36.85	37.69	31.38	
Missing	1,922	12.86	13.13	11.61	
CVD (%)					<0.001
No	14,056	89.75	92.06	78.88	
Yes	2,041	10.25	7.94	21.12	
Circadian syndrome (%)					<0.001
No	10,155	67.09	71.45	46.60	
Yes	5,943	32.91	28.55	53.40	

Diagnostic components of circadian syndrome (%)					<0.001
<4	10,155	67.09	71.45	46.60	
4	2,990	17.32	15.77	24.62	
5	2,355	12.84	11.09	21.09	
6	524	2.41	1.50	6.69	
7	74	0.33	0.19	1.00	
Central obesity (%)					<0.001
No	6,479	41.02	44.75	23.45	
Yes	9,619	58.98	55.25	76.55	
Elevated serum triglycerides (%)					<0.001
No	8,652	56.46	59.74	41.03	
Yes	7,446	43.54	40.26	58.97	
Reduced serum HDL-C (%)					<0.001
No	8,613	56.25	59.52	40.89	
Yes	7,485	43.75	40.48	59.11	
Hypertension (%)					<0.001
No	9,514	64.48	67.08	52.27	
Yes	6,584	35.52	32.92	47.73	
Elevated plasma glucose (%)					<0.001
No	6,445	43.90	46.72	30.63	
Yes	9,653	56.10	53.28	69.37	
Short sleep (%)					<0.001
No	13,805	88.05	88.95	83.82	

Yes	2,293	11.95	11.05	16.18	
Depression (%)					<0.001
No	14,669	92.37	94.30	83.31	
Yes	1,429	7.63	5.70	16.69	
UUI (%)					<0.001
No	12,195	78.79	87.78	36.52	
Yes	3,903	21.21	12.22	63.48	
UUI frequency (%)					<0.001
Never	12,195	78.82	87.82	36.54	
Less than once a month	1,710	10.04	7.52	21.91	
A few times a month	1,258	6.59	4.19	17.85	
A few times a week	563	2.79	0.47	13.70	
Every day and/or night	363	1.76	0.00	10.00	
Nocturia frequency (%)					<0.001
0	4,673	33.24	39.76	0.93	
1	5,925	39.65	46.06	7.88	
2	3,173	17.42	14.17	33.51	
3	1,490	7.11	0.00	42.36	
4	490	2.04	0.00	12.15	
5 or more	109	0.54	0.00	3.19	
OABSS (mean ± SE)		1.33 ± 0.02	0.87 ± 0.01	3.48 ± 0.02	<0.001

*For continuous variables, the t-test for slope was used in generalized linear models.

Values are expressed as mean+SD or %. SD=standard deviation, BMI = body mass index, PIR = poverty income ratio, HEI-2015= healthy eating index-2015, CVD = cardiovascular disease, UUI = urge urinary incontinence, OABSS = overactive bladder symptom score, eGFR = estimated

glomerular filtration rate.
